# Concentrating or Storing up Electricity

**Published:** 1881-07

**Authors:** 


### CONCENTRATING OR STORING UP ELECTRICITY.

Several years ago M. G. Planté, of France, made a secondary electrical battery, in which the electrical power of several ordinary cells could be concentrated or stored up within one cell, and the electrical force so gathered could be used when wanted. This battery consisted of two electrodes made of sheet lead, separated by strings of rubber, and placed in dilute sulphuric acid.

To charge this battery its poles were connected with an ordinary Bunsen or Daniell cell. During the operation of charging, one of the electrodes oxidizes, a brown coating of peroxide of lead soon showing itself thereon, and the metallic appearance disappears entirely; the other electrode also changes in appearance, its surface becoming powdery gray coating. When thus charged the secondary battery was capable of delivering an electric current of very much greater force than an ordinary cell of the same size. This secondary battery is capable of charge and discharge indefinitely. M. Faure has lately improved upon the Planté battery, by painting the lead sheets with red lead. Simple as the improvement is, the resulting effects are quite remarkable, the strong capacity and delivery of the battery being greatly increased. The chemical action that takes place is substantially the same as in the original Planté battery.

It is stated that one of M. Faure's secondary batteries, weighing 165 pounds, is capable of delivering a force equal to one horse power during the period of an hour. If this is so it would bring the weight of an electromotor and battery of one horse power within a gross weight of 200 pounds, and suggests, as one of the possibilities of the new discovery, the production of a carriage propelled by electricity, convenient and economical in use.

For the benefit of those who desire to try this interesting electrical contrivance, we give on another page an illustration in explanation of some recent impromptu experiments on the subject lately made in our office. Any intelligent person who has at hand a few sheets of lead may readily construct the new battery.

Professor Sir William Thomson, of Glasgow University, who has lately experimented with these new batteries, mentions the use of one of the cells, weighing 18 pounds, which Professor George Buchanan took with him in his carriage and successfully employed in removing a tumor from a child's tongue by heating a platinum wire. To have accomplished the same effect by the ordinary electrical means would have required the setting up of several voltaic cells, and involved much inconvenience. Professor Thomson anticipates that this method of storing electricity will have many practical uses. He speaks as follows:

" The largest useful application is waiting just now for the Faure battery, and I hope that a very minimum time will be allowed to pass until the battery supplied for this application is to do for electric light what a water cistern in a house does for an inconstant water supply. A little battery of seven boxes suffices to give the incandescence in the Swan or Edison lights to the extent of one hundred candles for six hours without any perceptible diminution of brilliancy. Thus, instead of needing a gas engine or steam engine to be kept at work as long as the light is wanted, with the liability of the light failing at any moment through the slipping of the belt or any other breakdown or stoppage of the machinery, and instead of the wasteful inactivity during the hours of the day or night, when the light is not needed, the engine may be kept going all day and stopped at night, or it may be kept going day and night, which undoubtedly will be the most economical plan when the electric light comes into general enough use.

"Another very important application of the accumulator is for the electric lighting of steamships. A dynamo-electric machine of very moderate magnitude and expense, driven by a belt from a drum on the main shaft, working through the twenty-four hours, will keep a Faure accumulator full, and thus, notwithstanding the irregularities of the speed of the engine at sea, or the occasional stoppages, the supply of electricity will always be ready to feed the Swan or Edison lamps in the engine rooms and cabins, or arc lights for the mast-head, and red and green side lamps, with more certainty and regularity than have yet been achieved in the gas supply for any house on *terra firma*. —*Scientific American*.

